# Rain Discrimination with Machine Learning Classifiers for Opportunistic Rain Detection System Using Satellite Micro-Wave Links

**DOI:** 10.3390/s23031202

**Published:** 2023-01-20

**Authors:** Christian Gianoglio, Ayham Alyosef, Matteo Colli, Sara Zani, Daniele D. Caviglia

**Affiliations:** 1 Department of Electrical, Electronics and Telecommunication Engineering and Naval Architecture (DITEN), University of Genova, 16145 Genova, Italy; 2 Artys, Darts Engineering Srl, 16121 Genova, Italy; 3 Consorzio Nazionale Interuniversitario per le Telecomunicazioni (CNIT), 43124 Genova, Italy

**Keywords:** satellite microwave links, oblique earth-space links, machine learning, smart rainfall system

## Abstract

In the climate change scenario the world is facing, extreme weather events can lead to increasingly serious disasters. To improve managing the consequent risks, there is a pressing need to have real-time systems that provide accurate monitoring and possibly forecasting which could help to warn people in the affected areas ahead of time and save them from hazards. The oblique earth-space links (OELs) have been used recently as a method for real-time rainfall detection. This technique poses two main issues related to its indirect nature. The first one is the classification of rainy and non-rainy periods. The second one is the determination of the attenuation baseline, which is an essential reference for estimating rainfall intensity along the link. This work focuses mainly on the first issue. Data referring to eighteen rain events were used and have been collected by analyzing a satellite-to-earth link quality and employing a tipping bucket rain gauge (TBRG) properly positioned, used as reference. It reports a comparison among the results obtained by applying four different machine learning (ML) classifiers, namely the support vector machine (SVM), neural network (NN), random forest (RF), and decision tree (DT). Various data arrangements were explored, using a preprocessed version of the TBRG data, and extracting two different sets of characteristics from the microwave link data, containing 6 or 12 different features, respectively. The achieved results demonstrate that the NN classifier has outperformed the other classifiers.

## 1. Introduction

Ever-increasing extreme weather events expose people and their assets to serious dangers. The absence of real-time information about the areal distributions of rainstorms at spatial scales that are in the order of the urban catchments and small basins causes environmental protection agencies not to know exactly where and how extreme rainfall will hit. Emergency phase management and flood risk require this crucial information for the short-term forecasting that constitutes the basic component of the decision support system. Developing a system for rainfall now-casting with a low cost and high temporal and spatial resolution by leveraging the latest technologies would lead to enhancing the prediction capabilities and efficiency of many infrastructures and environmental protection tools such as transport safety and flood warning systems. The traditional rainfall detection system consists of spaceborne remote sensing [1], weather radars [2], and rain gauges [3]. Recently, researchers have started leveraging the available radio spectrum sources to measure precipitation. The latest technique to predict precipitation takes advantage of existing commercial microwave links (CML). In [4], the authors presented a system that relied on a cellular network. The system provided reliable measurements for surface rainfall and, finally, they compared the estimated rainfall intensity of the proposed system with the measurements that came from the gauge. Horizontal microwave links (HML) have been utilized recently in many applications such as regional rainfall monitoring [5] and rain intensity inversion [6,7]. HMLs have the ability to monitor precipitation in urban areas [8] and they have formed an important part that supplements the present measurement method. Measuring precipitation can be performed using oblique earth-space links (OELs) [9]. In [10], authors used OELs to retrieve the rain intensity in Paris. A system for real-time rainfall estimation based on the measurements of the carrier to the ratio in the broadband satellite networks and artificial neural networks was proposed by [11]. Since OEL passes through the whole troposphere, there are more complicated factors that affect OEL compared to HML. Different atmospheric features such as turbulence, cloud, and gasses lead to two crucial issues in OELs. The first is the recognition of rainy and non-rainy periods rejecting variations in the signal intensity due to other factors, and the second is the determination of the signal baseline, i.e., the power level received with clear skies, from whose value it is possible to calculate the amount of attenuation induced by the presence of rain [12]. The importance of the second issue comes from the fact that determining the baseline is crucial for getting the attenuation produced by rainfall. The authors of [13] proposed a method for estimating the baseline based on the minimal attenuation values. Such a method does not require the classification of the rainy and non-rainy periods. The interpolation for the signal before and after precipitation has been used to obtain the attenuation baseline for the rainy periods in many works [14,15]. The slow Kalman tracker provides the last dry baseline and it is used as an initial reference for baseline determination [16]. Many researchers have utilized machine learning techniques to solve the first issue, and those techniques outperformed the traditional ones. A method for identifying the rainy period depending on a metric related to the receiver bit error rate parameter which is provided by satellite receivers has been implemented by Adirosi et al. [17]. An approach for identifying whether a given period is rainy or not using long short-term memory (LSTM) architecture has been proposed by [18]. They first extracted the features from the received signals and then trained an LSTM network to detect whether the period is rainy or not. Artificial neural networks (ANN) have also been used to recognize the rainy and non-rainy periods by [10,19] and convolutional neural networks (CNN) by [20]. The classification of rainy and non-rainy periods has been performed using Markov switching models [21] and by analyzing the signal in the frequency domain by applying Fourier transformations on a rolling window [22]. Deciding whether a given period is rainy or not can be defined as a binary classification problem. Different classifiers have been used in previous works for the classification of rainy conditions from microwave links [10,13,18,23], such as SVM and LSTM classifiers. In this work, four shallow machine learning algorithms have been proposed and compared to find out the most suitable one for classifying rainy and non-rainy observations from satellite down-links signals. The goal is to identify an effective classifier being also energy efficient to be deployed in a low-power and low-cost embedded device supplied by a battery. The four algorithms are an ANN with one hidden layer, a kernel-support vector machine (K-SVM), a decision tree (DT), and a random forest (RF). The performances of the classifiers have been compared by evaluating three metrics: specificity indicating the capability of classifying non-rainy samples, recall measuring the correctness in predicting the rainy samples, and F1-score averaging the precision (ratio between true rainy and classified as rainy samples) and the recall.

The remainder of the paper is organized as follows: Section 2 presents the dataset and its processing, Section 3 describes the algorithms and the metrics computed to evaluate the performance, Section 4 reports the results and discussion, and conclusions are drawn in Section 5.

## 2. Problem Definition

This section describes the dataset employed in this work, the events selected from the datasets used to train and test the classifiers, and the features extracted from the raw data.

### 2.1. Data Collection

The OEL data that has been used in this work have been collected from the smart rainfall system (SRS) which is a network of microwave sensors for satellite down-links developed by the University of Genoa and Artys, Darts Engineering Srl, Genoa, Italy [24]. The analysis is focused on measurements performed by an SRS sensor installed on the roof of the University of Genoa—DITEN department, characterized by an offset antenna with a parabolic reflector with a diameter of 85 cm, and aligned towards the Turksat 42° E Constellation. The azimuth and elevation angles of the dish with respect to the sun are $137.09^{\circ }$ and $29.2048^{\circ }$, respectively. The sensor was programmed to receive the signal in the upper Ku band (11.7∼12.75 GHz) with vertical polarization [25]. A universal low noise block (LNB), placed on the arm of the parabolic dish, converts the microwave signals from the Ku-band to the L-band. The LNB interface circuit provides the power supply and the 22 kHz tone to select the proper sub-band (10.75 to 11.7 GHz and 11.7 to 12.75 GHz) and polarization. The RF signal, after filtering and amplification, is sent to a detector that converts the power over a 45 dB range into a voltage using logarithmic amplifiers. The LNB interface board consists of a microcontroller that also performs the signals analog-to-digital conversion (ADC) with 10-bit resolution. The microcontroller samples the RF signals 64 times per minute; at the end of each minute, a UDP packet including the input RF power measurement and some information is sent to a central server for further processing. The block diagram of the system is reported in Figure 2 of [26], while the SRS sensing board is shown in Figure 3 [26].

**Figure 1:**
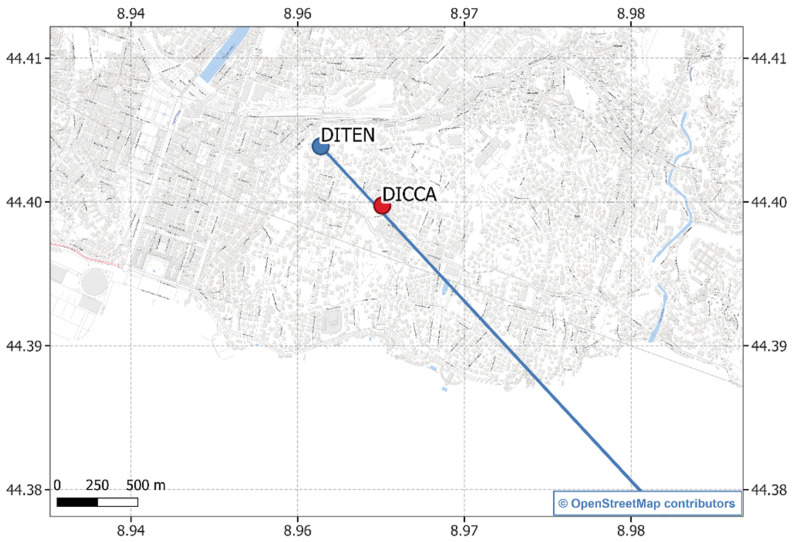
The location of the SRS dish at the University of Genova—DITEN department (blue circle, Lat: 44.4031; Lon: 8.9587; Altitude 70 m.a.s.l.) and TBRG at the Hydraulic Laboratory of the University of Genova—DICCA department (red circle, Lat: 44.3998; Lon: 8.9636; Altitude 45 m.a.s.l.). The blue line represents the ground projection of the path from the dish toward the Turksat 42° E constellation, chosen for this research.

The ground data used as a reference for actual rainfall occurrence were obtained by a dynamically calibrated TBRG manufactured by CAE S.p.A.—San Lazzaro di Savena, Italy—and installed at the Hydraulic Laboratory of the University of Genova—DICCA department [25]. Figure 1 shows a cartographic overview of the position of the dish and the reference rain gauge (TBRG) where the blue circle on the map is the location of the SRS dish, and the red circle is the location of the TBRG.

**Figure 2:**
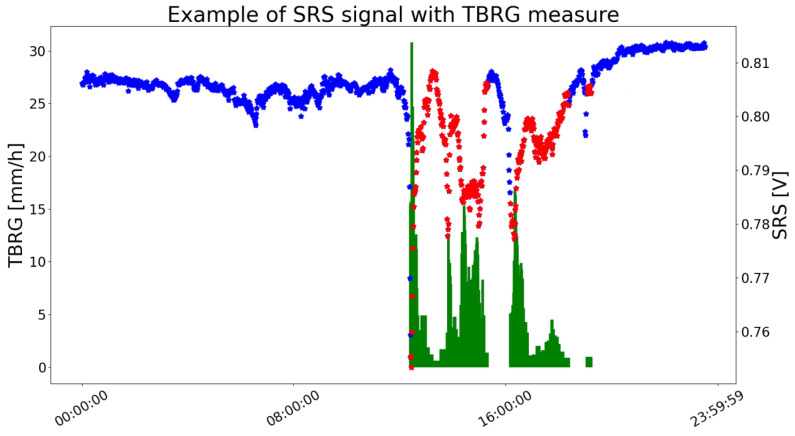
Example of one day signal collected by the SRS system with the TBRG reference. The histogram in green represents the measurement of the TBRG in mm/h. The dots represent the readings from the SRS system: they are marked in blue when the TBRG output is zero, and they are marked in red otherwise.

The choice of the instrument providing reference measurement was made considering the high level of accuracy of the DICCA’s TBRG, since it has been corrected from the systematic mechanical errors reaching class A performance (according to EN 17277:2019 recommendations) and constantly monitored by the WMO Lead Centre “B. Castelli” on Precipitation Intensity laboratory. Figure 4 in [25] provides the percentage relative error as a function of the rainfall intensity. The error is in the range ±3% for all the rainfall measurements. Furthermore, its location (Figure 1) is below the SRS microwave down-link considered in this study, at a distance of approximately 500 m from the dish, and is therefore suitable for providing a reasonable indication of the rainfall conditions occurring in the monitored territory. To improve the reference dataset quality, the inter-tip time correction method described by Colli et al. [27] was applied. This processing technique is able to provide more accurate one-minute rainfall measurements, with respect to the coarse method yet very common in operational practice, i.e., the counting of the number of tips performed by the TBRG mechanical sensor over a specified measurement update interval. Such a number is usually associated with a rainfall volume, given the TBRG sensitivity and calibration parameters. The datasets for both the SRS dishes and the TBRG contain measurements collected from April 2017 to April 2019 and are publicly available (https://github.com/cosmiclabunige/Rainfall_Prediction_18_days (accessed on 18 January 2023)). Each day contains 1440 observations, i.e., one for each minute. Figure 2 provides an example of a daily signal collected by the SRS system and the reference measured by TBRG: in blue the SRS signal, in green the TBRG measure indicating the intensity of rainfall in each minute, in red the SRS observations in which a rainfall happened.

### 2.2. Event Selection

Deciding whether a given observation is rainy or not has been performed using a thresholding criterion on the TBRG measures for the corresponding sample. The threshold has been set to 0.1 mm/min. In this way, it is possible to label the dataset: a sample that presents a TBRG measure lower than the threshold is labeled as not-rain, while if the TBRG presents a value greater or equal to 0.1 mm/min the sample is labeled as rain. This threshold has been selected for consistency with the sensing capabilities of common tipping bucket type rain gauges which in general have a minimum sensitivity that is higher or equal to 0.1 mm [28]. We considered as interesting events for our research the days during which the rain occurred with maximum intensity in one minute greater than 15 mm/h or a cumulative in the whole day off at least 50 mm. Table 1 shows the details of the 18 events that have been considered in this work in terms of maximum rainfall intensity Max(RI) [mm], total rainfall accumulation Htot [mm/h] in 60 min, and the number of rainy minutes in each day. It is worth noting that the events are in general strongly unbalanced, having a number of minutes with rainfall much lower than non-rainy observations, except for event 10.

### 2.3. Description of the Feature Set

Similarly to a recent work proposed in the literature [12], 12 statistical features with a fixed time window were extracted from the SRS signal, in addition to the class label that represents whether the given moment is rainy or not, derived from TBRG data. This creates the dataset for this work. Table 2 shows the description of the dataset features with their corresponding time window. After computing the features and in order to reduce the amount of time needed for the training phase [29], the Min-Max Scaler has been implemented on the features to fall within the range [0, 1] with the following formula:

(1) $$x_{normalized}=\frac{x-x_{min}}{x_{max}-x_{min}}$$

where $x_{max}$ represents the maximum value of the feature and $x_{min}$ represents the minimum value of the feature.

## 3. Methodology

The methodology followed for training and testing was inspired by a leave-one-out approach [30]: one day among the 18 selected has been chosen for testing and the remaining 17 for training; iteratively, all 18 days have been tested in this way. For each of the 18 problems defined so far, each having a total of 24,480 data for training, the training dataset has been balanced by taking all the rainy data and extracting randomly the same number of non-rainy data. A total of 20% of the training data was used as the validation set for the tuning of the hyperparameters of the four algorithms. This was performed using the grid search technique [31] applied to possible pools of candidates, seeking the configuration that led to the best accuracy on the validation set.

### 3.1. Machine Learning Algorithms with Hyper-Parameters Selection

The definitions of the classifiers utilized in the work are reported below with the list of hyperparameters tuned during the training phase. The choices of the algorithms and their hyperparameters have been made in the perspective of the future deployment of the classification algorithms on a device with limited computational resources and powered by an energy harvester (e.g., a solar panel). In fact, to keep operating costs, i.e., both data transfer and energy consumption, to a minimum, a real-time monitoring system with sensors distributed across the territory requires that the classification be performed next to the sensors (on the edge). In this way, the sending of data to the server of the operations center takes place only when precipitation events occur. Consequently, the algorithms have to be compliant with the hardware constraints of the devices; moreover, the computational cost was measured as the inference time and the energy consumption must be as low as possible. In addition, it is worth considering, as regards the ML algorithm itself, e.g., artificial neural networks, that the greater the complexity of the classifier (higher number of parameters), the higher the risk of overfitting the training set. Hence, in that case, the classifier will perfectly predict the data belonging to the training set but misclassify the new data, featuring low generalization performance. It was therefore our aim to restrain the complexity of the algorithms considered.

#### 3.1.1. Decision Tree

DT is a machine learning technique where the data is divided constantly according to a certain parameter. The tree has two types of entities which are leaves and decision nodes. The leaves represent the output and the decision nodes represent the split location [32]. The hyperparameters pool considered during the training procedure of DT is as follows:

- Maximum depth of the tree, max_depth=[4,8,None] where None means no limit.
- Minimum number of samples to split a node, min_samples_split=[2,5,10].
- Number of samples to consider a node as a leaf, min_samples_leaf=[1,5,10].

#### 3.1.2. K-SVM

SVM is a machine learning technique whose output is a hyperplane in M dimensional space where M represents the number of features. The output hyperplane is the best hyperplane that can separate the data points according to their classes [33]. The hyperparameters pool considered during the training procedure of SVM is as follows:

- The Kernel used is RBF.
- Standard deviation of the RBF kernels, $Gamma=[10^{i},for\;\;i\;in\;range\left(-4,4\right)]$.
- L2 Regularizer, $Lambda=[10^{i},for\;\;i\;in\;range\left(-4,4\right)]$.

#### 3.1.3. Artificial Neural Network

ANNs are designed in a way that simulates the human brain and how it processes and analyzes information. ANNs are able to solve problems that are difficult for both humans and statistical methods. With more data fed to ANNs, the ANNs can give much better results. The selected architecture for this work was the multilayer perceptron, while the Adam optimizer, which implements a version of the gradient descent algorithm [34], has been used for its training. The hyperparameters pool considered during the training procedure of ANN is as follows:

- Number of neurons, N=[50,100,200,300].
- L2 Regularizer $Lambda=[10^{i},for\;\;i\;in\;range\left(-4,4\right)]$.

#### 3.1.4. Random Forest

RF is a set of a large number of independent decision trees that works in a way where each individual tree outputs a class prediction and the final class prediction of the RF is taken as a vote for the most predicted class by the individual trees. The hyperparameters pool considered during the training procedure of Random Forest is as follows:

- Number of trees, num_trees=[50,75,100].
- Maximum depth of the tree, max_depth=[4,8,None] where None means no limit.
- Minimum number of samples to split a node, min_samples_split=[2,5,10].
- Number of samples to consider a node as a leaf, min_samples_leaf=[1,5,10].
- Percentage of random training data to create a tree, max_samples=50%.

### 3.2. Performance Metrics

Three metrics have been computed to assess the performance of the classifiers. Since the 18 tested datasets are highly unbalanced, the accuracy has not been computed because it would have been a biased metric and thus not represented a fair indicator of the classifiers’ goodness. For ease of use, the following notations are used: true positives (TP) are rain minutes correctly classified, false positives (FP) are non-rain minutes incorrectly classified as rain, true negatives (TN) are non-rain minutes correctly classified, false negatives (FN) are rain minutes incorrectly classified as non-rain. The three metrics are listed in the following:

1. Specificity is the ratio between the TN and all the negatives, indicating how good a classifier is in predicting non-rainy observations.
  (2) $$Specificity=\frac{TN}{TN+FP}$$
2. Recall is the measure of our model correctly identifying TP. Thus, for all the samples that actually are rainy, recall tells us how many of them the classifier has correctly identified as rainy samples.
  (3) $$Recall=\frac{TP}{TP+FN}$$
3. F1-score represents the Harmonic mean of the precision and recall. The precision is the ratio between the TP and all the positives. For our problem statement, that would be the measure of samples that the classifier has correctly identified as rainy samples out of all the samples that have been predicted as rainy by the classifier.
  (4) $$F1\text{-}Score=\frac{2*Precision*Recall}{Precision+Recall}$$

## 4. Results and Discussion

In this section, Table 3 provides the classification results obtained for using the whole set of 12 features. The considered algorithms are, in order, the DT, KSVM (KS in the Tables), NN, and RF. The results are described in terms of the specificity, recall, and F1-Score using the improved TBRG as a reference and after applying the leave-one-out approach where in each round one day was selected for testing and the remaining ones were used for training. The last two rows of the table represent the average and the standard deviations of the metrics on the 18 days. Table 4 reports similar results, obtained in this case using the reduced set of six features (from x1 to x6).

**Figure 3:**
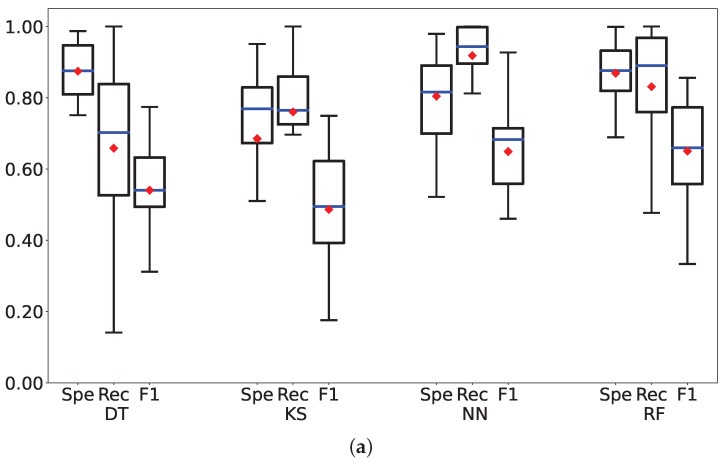

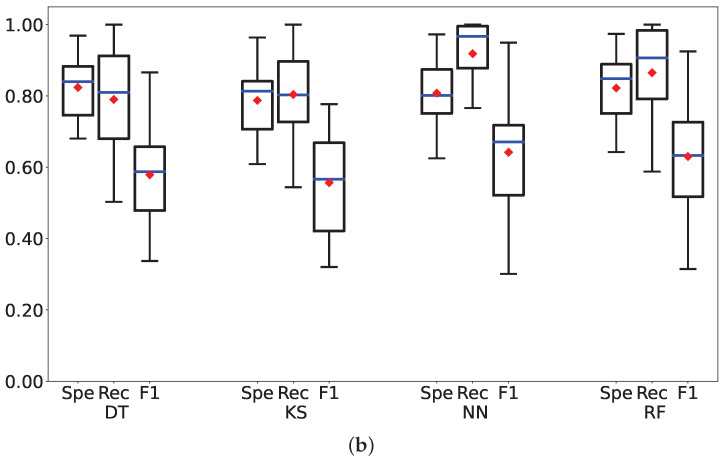
For each classification algorithm (from left to right DT, KS, NN, RF) trained with 12 features (top: (**a**)) and 6 features (bottom: (**b**)) a triplet of boxplots is shown, reporting the specificity (Spe), recall (Rec), and F1-score (F1) metrics, respectively. (**a**) Boxplot of the metrics using 12 Features. (**b**) Boxplot of the metrics using 6 Features.

Figure 3 presents the boxplots of the metrics shown in Table 3 and Table 4. The lower and upper parts of the boxes delimit the first and third quartiles, while the whiskers mark the variability outside the quartiles. A red diamond symbol represents the average value of each metric, while blue horizontal lines represent the median values, respectively.

By looking at the classification performance reported in Table 3 and in the associated Figure 3a, we can notice that NN and RF present the best trade-off among the three metrics. NN outperforms RF in the recall metric, meaning that it is better at classifying rainy observations, while RF achieves a higher specificity, i.e., it classifies the non-rainy data more accurately. DT has the highest specificity but the lowest recall, thus it is not suitable for the classification of rainfall. Observing the F1-Score results, which represent the harmonic mean for precision and recall, it is noticeable that the RF and NN classifiers perform better than the DT and KSVM classifiers. An important comment should be raised, explaining that if the rain gauge detects rainfall, it is a sufficient condition for a rain occurrence over the SRS link, while the opposite is not true (i.e., if the rain gauge does not detect rainfall, it can occur elsewhere over the link, due to its extension). Consequently, the specificity metric, which gains lower levels, is less meaningful than the recall one, which actually presents very good values. Moreover, it is worth noting that adopting a subset of features (i.e., six features in this work), NN and RF still present the best trade-off with respect to the other classifiers.

In particular, the average recall of NN is equal to the one obtained by using all 12 features, but the variability of the results is slightly higher. Meaning a small increase in the misclassification rate of rainy observations. On the other hand, the variability of NN specificity is smaller, with an average value of 1% better than by adopting 12 features. The specificity of RF decreases when using six features, while the recall increases. In general, reducing the number of features and maintaining a reasonable quality of the results, is very important within the machine learning approach, leading in fact to less complex models with short processing time and smaller storage capacity needs. This allows for reducing response time, which is a very crucial aspect of rainfall monitoring systems, in particular from the perspective of the implementation of such algorithms according to the edge computing paradigm. Based on the classification results so far obtained, we can identify the NN as the best classifier since it outperformed the DT, KSVM, and RF classifiers.

**Figure 4:**
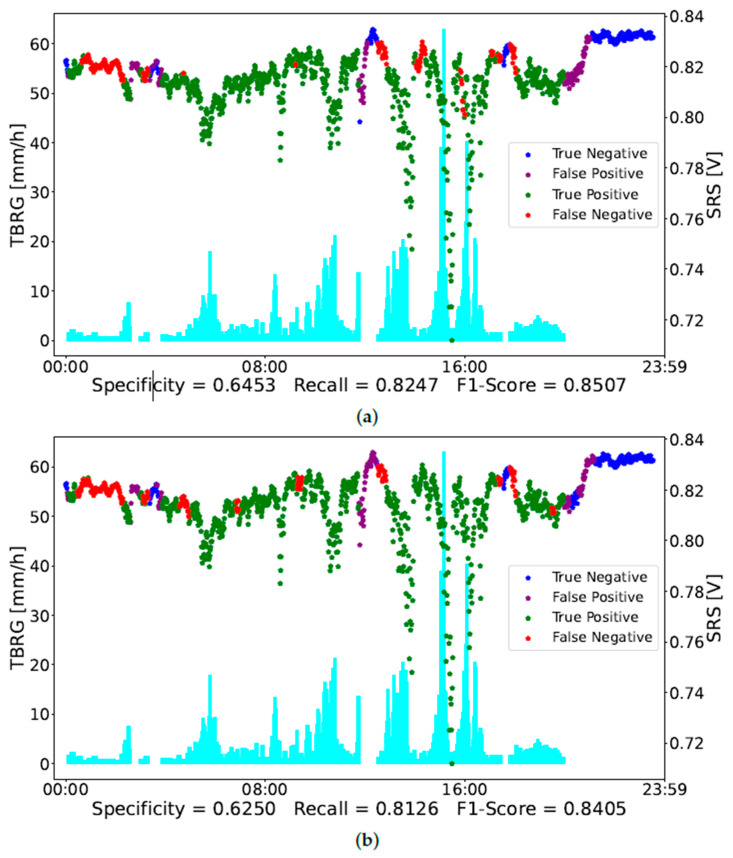
Day 11 classified by NN trained with 12 features (**a**) and with 6 features (**b**). The blue dots refer to not-rainy observations correctly classified, the purple ones are not-rainy observations classified as rain, the green ones are rainy observations correctly classified, and the red dots represent rainy observations classified as not rain. The cyan bars represent the TBRG measurements different from 0 mm/h, i.e., associated with the rain observations. (**a**) Day 11 Neural Network 12 Features. (**b**) Day 11 Neural Network 6 Features.

**Figure 5:**
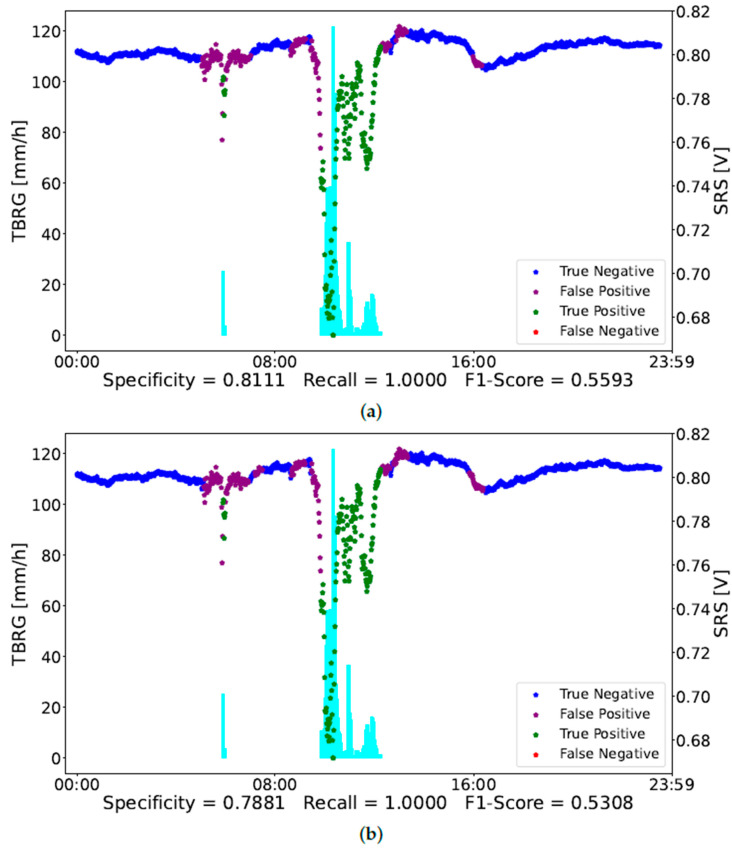
Day 17 classified by NN trained with 12 features (**a**) and with 6 features (**b**). The blue dots refer to not-rainy observations correctly classified, the purple ones are not-rainy observations classified as rain, the green ones are rainy observations correctly classified, and the red dots represent rainy observations classified as not rain. The cyan bars represent the TBRG measurements different from 0 mm/h, i.e., associated with the rain observations. (**a**) Day 17 Neural Network 12 Features. (**b**) Day 17 Neural Network 6 Features.

Figure 4 and Figure 5 report an example of the classification of two days of observations, i.e., day 11 and day 17 in Table 1, respectively. In Figure 4, the red dots, representing a misclassification of rain conditions, correspond to a low intensity of rain measured by the TBRG. On the other hand, the more intense rainy observations are well-classified by the NN. The purple dots point out a misclassification in non-rainy observations. As mentioned above, specificity could be not that accurate since the rain gauge is not close to the SRS. Indeed, the misclassified observations occurring around 12:00 seem to be related to a rain event that has not been detected by the TBRG.

In Figure 5, the NN classified correctly all the rain observations, thus having a recall of 100%. On the contrary, it misclassifies some non-rainy observations. It is worth noting that most of these points occur near the rain observations. Probably some of them refer to a rain condition not measured by the TBRG.

## 5. Conclusions

In this work, four different machine learning-based classification techniques were applied to classify rainy and non-rainy periods on different events using a rain intensity threshold of 0.1 mm/min. The features for the dataset have been extracted from the data obtained from a smart rainfall system (SRS) installation, with a dish diameter of 85 cm pointed to the Turksat 42° E constellation. Reference rain measurements from an improved TBRG have been used to obtain the class label for this work. Eighteen days of observations were collected in a dataset, using iteratively one day for testing and the remaining seventeen for the training of four algorithms. From the SRS signals, 12 features were extracted, and a reduced subset (i.e., 6 features) for the sake of comparison has been selected. The results show that the best trade-off evaluated on the 18 days in terms of specificity, recall, and F1-score, is achieved by the neural network classifier for both the feature sets. This work paves the way for developing a fully automatic real-time rainfall monitoring system, with a dense population of sensors deployed over the territory, where the rain discrimination algorithm is hosted at the edge, by devices with constrained resources, both on the computational and the energy requirements point of view. In this way, it will be possible to reduce the data transfer to the server on the cloud, as well as the cost of the service, to the essentials, just when rainfall occurs. Further developments will concern the deployment of the classification algorithm on an embedded device for the continuous monitoring of precipitations. Moreover, for continuous rainfall monitoring and because of the seasonality of the data, it will be necessary to fine-tune the classifier online after a period of observations.

## Figures and Tables

**Table 1:** Selection of rainfall events and overall quantities as measured by the DICCA TBRG.

Event ID	Date	Max TBRG [mm]	Htot [mm/h]	Minutes of Rainfall
1	3 May 2017	83.9	15.2	106
2	6 May 2017	30.8	24.4	319
3	11 July 2017	218	22.4	61
4	22 July 2017	111.3	32.4	99
5	9 September 2017	146.8	52.1	328
6	18 September 2017	111.4	19.4	262
7	4 November 2017	65.9	9.2	73
8	5 November 2017	139.5	47.4	386
9	25 November 2017	43.4	21.0	342
10	10 December 2017	12.7	17.6	393
11	11 December 2017	62.9	65.0	1081
12	25 December 2017	54.5	7.1	61
13	26 December 2017	26.7	15.1	259
14	27 December 2017	58.7	42.9	481
15	1 January 2018	50.5	32.4	369
16	27 January 2018	24.9	19.4	295
17	14 August 2018	121.7	35.5	151
18	4 April 2018	220.4	38.5	346

**Table 2:** Description of the extracted statistical features from the satellite-to-earth microwave signal.

Symbol	Time Window with Respect to the Given Moment	Feature
x1	30 min before	Average
x2	30 min before	Standard Deviation
x3	30 min before	Maximum
x4	30 min before	Minimum
x5	30 min before	Skewness
x6	30 min before	Kurtosis
x7	30 min before	Local Trend
x8	30 min before	Information Entropy
x9	30 min before	Ratio of Singular Values
x10	30 min before	Ratio of High Frequency Energy to Low
x11	none	Probability higher than Standard Deviation
x12	none	Probability higher than Average

**Table 3:** Classification results by algorithms using 12 features.

ID	Specificity				Recall				F1-Score			
	**DT**	**KS**	**NN**	**RF**	**DT**	**KS**	**NN**	**RF**	**DT**	**KS**	**NN**	**RF**
1	0.87	0.90	0.89	0.92	0.84	0.72	0.97	1.0	0.49	0.48	0.58	0.67
2	0.91	0.92	0.89	0.94	0.70	0.76	1.0	0.91	0.70	0.75	0.84	0.86
3	0.97	0.95	0.92	0.90	1.0	0.77	0.97	1.0	0.73	0.54	0.51	0.49
4	0.89	0.51	0.89	0.90	1.0	1.0	0.96	1.0	0.49	0.18	0.46	0.51
5	0.96	0.67	0.52	0.87	0.40	0.78	1.0	0.80	0.52	0.54	0.56	0.71
6	0.77	0.80	0.79	0.77	0.93	0.86	1.0	0.98	0.63	0.63	0.69	0.66
7	0.86	0.83	0.64	0.82	0.29	0.85	1.0	0.88	0.15	0.34	0.23	0.33
8	0.87	0.82	0.72	0.85	0.62	0.76	0.92	0.69	0.63	0.68	0.69	0.66
9	0.75	0.70	0.66	0.76	0.60	0.47	0.89	0.75	0.50	0.39	0.60	0.60
10	0.99	0.68	0.98	1.0	0.19	0.28	0.57	0.29	0.31	0.26	0.70	0.45
11	0.77	0.74	0.65	0.69	0.61	0.52	0.82	0.77	0.73	0.65	0.85	0.82
12	0.96	0.92	0.97	0.97	0.84	0.90	0.85	0.89	0.63	0.48	0.68	0.70
13	0.80	0.70	0.69	0.71	0.73	0.82	0.92	0.90	0.54	0.51	0.55	0.55
14	0.89	0.81	0.87	0.86	0.77	0.75	0.93	0.93	0.77	0.70	0.84	0.84
15	0.97	0.29	0.97	0.98	0.14	0.75	0.93	0.76	0.23	0.40	0.93	0.84
16	0.85	0.29	0.80	0.89	0.71	1.0	0.99	0.94	0.62	0.43	0.72	0.79
17	0.77	0.00	0.81	0.83	0.99	1.0	1.0	1.0	0.51	0.19	0.56	0.59
18	0.88	0.80	0.82	0.99	0.50	0.70	0.81	0.48	0.54	0.60	0.69	0.63
**Avg**	0.87	0.69	0.80	0.87	0.66	0.76	0.92	0.83	0.54	0.49	0.65	0.65
**Std**	0.08	0.26	0.13	0.09	0.27	0.19	0.11	0.19	0.17	0.17	0.17	0.15

**Table 4:** Classification results by algorithms using 6 features.

ID	Specificity				Recall				F1-Score			
	**DT**	**KS**	**NN**	**RF**	**DT**	**KS**	**NN**	**RF**	**DT**	**KS**	**NN**	**RF**
1	0.87	0.82	0.85	0.89	0.93	0.86	0.98	0.99	0.52	0.42	0.51	0.59
2	0.87	0.84	0.92	0.88	0.83	0.79	0.94	0.86	0.73	0.67	0.84	0.76
3	0.88	0.86	0.87	0.92	0.93	10.0	10.0	10.0	0.42	0.40	0.41	0.52
4	0.84	0.84	0.88	0.87	10.0	10.0	10.0	10.0	0.39	0.39	0.46	0.45
5	0.68	0.64	0.71	0.69	0.85	0.84	0.99	0.96	0.58	0.55	0.67	0.65
6	0.79	0.80	0.78	0.78	0.80	0.77	10.0	0.89	0.59	0.58	0.67	0.62
7	0.79	0.78	0.75	0.76	10.0	0.95	10.0	10.0	0.34	0.32	0.30	0.31
8	0.79	0.75	0.76	0.80	0.75	0.76	0.90	0.77	0.65	0.63	0.71	0.67
9	0.69	0.69	0.67	0.69	0.61	0.54	0.78	0.63	0.47	0.43	0.56	0.48
10	0.95	0.92	0.91	0.93	0.56	0.65	0.63	0.59	0.66	0.70	0.68	0.67
11	0.73	0.66	0.63	0.64	0.61	0.68	0.81	0.72	0.72	0.76	0.84	0.78
12	0.97	0.96	0.97	0.97	0.74	0.90	0.87	0.92	0.61	0.67	0.70	0.74
13	0.73	0.65	0.65	0.66	0.82	0.82	0.92	0.90	0.53	0.47	0.52	0.52
14	0.88	0.81	0.85	0.88	0.79	0.88	0.95	0.92	0.78	0.78	0.85	0.85
15	0.96	0.86	0.96	0.97	0.85	0.72	0.99	0.93	0.87	0.68	0.95	0.92
16	0.88	0.84	0.80	0.83	0.66	0.76	0.99	0.87	0.62	0.65	0.72	0.70
17	0.69	0.61	0.79	0.75	0.99	10.0	10.0	10.0	0.43	0.38	0.53	0.49
18	0.84	0.83	0.80	0.86	0.50	0.57	0.77	0.63	0.51	0.55	0.65	0.62
**Avg**	0.82	0.79	0.81	0.82	0.79	0.80	0.92	0.87	0.58	0.56	0.64	0.63
**Std**	0.09	0.10	0.10	0.10	0.15	0.14	0.11	0.14	0.14	0.14	0.11	0.15

## Data Availability

Data are publicly available at: https://github.com/cosmiclabunige/Rainfall_Prediction_18_days (accessed on 18 January 2023).
